# Formation of Membrane Domains via Actin Waves: A Fundamental Principle in the Generation of Dynamic Structures in Phagocytes

**DOI:** 10.3390/ijms26104759

**Published:** 2025-05-16

**Authors:** Jiro Takito, Naoko Nonaka

**Affiliations:** Department of Oral Anatomy, School of Dentistry, Showa Medical University, 1-5-8 Hatanodai, Shinagawa, Tokyo 142-8555, Japan; choku@dent.showa-u.ac.jp

**Keywords:** bone resorption, macrophages, osteoclasts, phagocytosis, self-organized actin wave

## Abstract

Phagocytes carry out their functions by organizing new subcellular structures. During phagocytosis, macrophages internalize and degrade pathogens and apoptotic cells by forming the phagocytic cup and phagosome. Osteoclasts resorb bone by forming the sealing zone and ruffled border at the ventral membrane. This review explores the organizational principles of these dynamic structures. In in vitro frustrated phagocytosis, specifically 2D phagocytosis by macrophages, the activation of the Fcγ receptor generates multiple self-organized waves containing F-actin, Arp2/3, and phosphoinositides. The propagation of these circular actin waves segregates the inside from the outside, leading to the compartmentalization of the ventral membrane. As the actin wave passes, cortical actin is disrupted, and membrane remodeling occurs within the wave, creating a new membrane domain with high exocytic activity. These processes mirror the formation of the constriction zone in the phagocytic cup and phagosome during 3D phagocytosis. A similar mechanism may also contribute to the formation of the sealing zone and ruffled border in osteoclasts. Based on these observations, we propose that dynamic structures formed from actin waves are organized through the fractal integration of self-organized, oscillatory substructures, with F-actin treadmilling fueling their formation and maintenance.

## 1. Introduction

Professional phagocytes, including macrophages, neutrophils, and dendritic cells, degrade pathogens and apoptotic cells through phagocytosis [1,2,3]. Phagocytosis is a key component of the innate immune system, aimed at eliminating harmful substances from the body. The process is divided into three main stages: detection, internalization, and degradation of the target. Each step involves specific subcellular structures, such as the phagocytic receptor, phagocytic cup, and phagosome. Macrophages possess a variety of receptors, including opsonic and non-opsonic receptors, to identify phagocytic targets [4]. Ligand–receptor binding induces receptor clustering and activates downstream signaling cascades, which lead to the reorganization of the actin cytoskeleton and local membrane remodeling, ultimately forming the phagocytic cup. After target internalization, the phagocytic cup fuses at its rim and evolves into the phagosome, a new organelle. The phagosome becomes a digestive organelle through the vigorous transport of lysosomal enzymes. Once the target is degraded into harmless constituents, the phagosome undergoes fragmentation, vesicle budding, tubulation, and constriction [5,6]. Macrophages continually repeat this cycle throughout their lifespan.

Although bone appears to be an inert connective tissue composed primarily of hydroxyapatite, it is continuously regenerated through a process called bone remodeling, where old bone is replaced with new bone. This process renews approximately 10% of the skeleton each year in healthy adults [7]. Bone remodeling occurs within specialized areas known as Basic Multicellular Units (BMUs) [8,9], which have a lifespan of 6–9 months. At any given time, around one million BMUs are active in the body. Each BMU consists of osteoclasts at the front, osteoblasts at the rear, and a central vascular capillary. Osteoclasts degrade old bone, while osteoblasts form new bone. The activities of osteoclasts and osteoblasts are tightly regulated to maintain bone homeostasis, and disturbances in this balance are often linked to bone diseases. Although bone is considered “self” tissue, osteoclasts recognize and degrade damaged or aged bone, suggesting the existence of an unknown mechanism that triggers this process [10,11]. Osteoclastic bone resorption occurs in an extracellular space formed by the sealing zone, ruffled border, and the bone surface [12,13]. The sealing zone is an actin ring composed of F-actin and podosomes, while the ruffled border is a highly convoluted membrane domain within the sealing zone. Osteoclasts attach firmly to the bone via the sealing zone and secrete protons and proteases through the ruffled border into the resorption space. While the roles of these subcellular structures in bone resorption are well recognized, the mechanisms behind their formation and maintenance remain unclear. We have previously proposed that the self-organized actin wave is the origin of the sealing zone [14].

Before comparing macrophage phagocytosis with osteoclastic bone resorption, we first provide an overview of the two cell types. Both macrophages and osteoclasts are derived from the same monocyte/macrophage lineage, but macrophages are mononucleated, while osteoclasts are multinucleated giant cells [15]. Macrophages play a central role in innate immunity. Unexpectedly, osteoclasts are also involved in immune responses [16,17]. Osteoclasts function as antigen-presenting cells and activate CD4^+^ and CD8^+^ T cells [18]. In vitro, osteoclasts are capable of phagocytosing latex and polymethylmethacrylate particles [19] and apoptotic bone cells [20]. On the other hand, alveolar macrophages do not resorb bone, though macrophages differentiated into osteoclast-like cells can resorb bone in vitro [21]. Thus, while macrophages and osteoclasts differ in several aspects, the processes of phagocytosis and bone resorption are distinct cellular events. However, understanding the formation of the 2D phagocytic cup during frustrated phagocytosis could provide new insights. This knowledge offers an organizational principle for the formation of the phagocytic cup and phagosome during phagocytosis. In this review, we first introduce the process of 2D phagocytic cup formation mediated by self-organized actin waves. We then summarize the properties of these divergent waves with a focus on subcellular morphogenesis. Finally, we return to the bone resorption apparatus in osteoclasts and discuss the likely organization of oscillatory phagocytic structures.

## 2. Phagocytic Cup in Macrophage Phagocytosis

**Figure 1:**
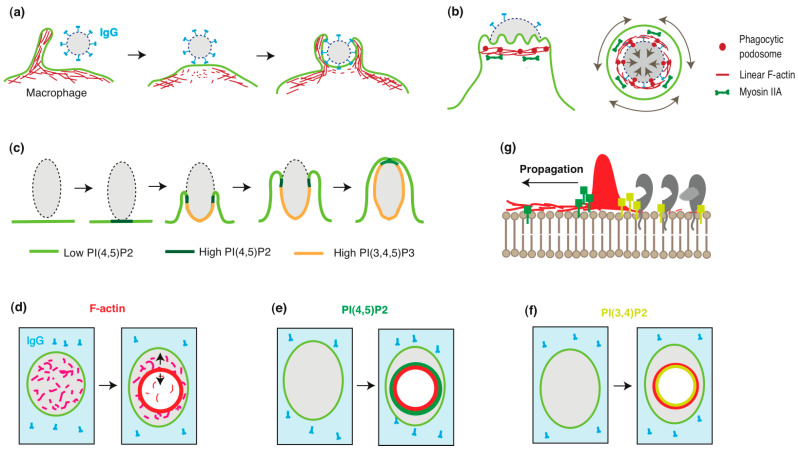
**Macrophage phagocytosis involves the compartmentalization and remodeling of the plasma membrane.** (**a**) Disruption of the cortical actin during phagocytic cup formation. Macrophages probe for phagocytic targets using filopodia or ruffles. The binding of IgG (blue) to FcγR triggers signals that locally disrupt the actin cortex (red) at the base of the emerging phagocytic cup. Adopted and modified from [1]. (**b**) The phagocytic cup tightly grasps the target via the constriction zone. Side view (left) and cross-section (right) of the constriction zone. F-actin accumulates near the front of the phagocytic cup, colocalizing with maximal inward forces, regardless of the engulfment stage. The constriction zone comprises linear F-actin cables (red lines) and phagocytic podosomes (red circles). The cables form a contractile ring, generating circumferential force via interactions with myosin II (green). The podosomes protrude against the target, exerting force through Arp2/3-mediated F-actin polymerization. Adopted and modified from [28]. (**c**) Dynamic localization of phosphoinositides during phagocytic cup formation. PI(4,5)P_2_ (yellow-green) is uniformly distributed on the ventral membrane of the macrophage. Upon IgG stimulation, PI(4,5)P_2_ transiently increases near the stimulation site (green), followed by its depletion and a concurrent increase in PI(3,4,5)P_3_ (yellow) in the phagocytic cup. The PI(4,5)P_2_ ring persists until the phagocytic cup seals. Adopted and modified from [29]. (**d**–**g**) Actin wave expansion and membrane remodeling during frustrated phagocytosis. (**d**) On IgG-coated surfaces (blue), macrophages generate propagating actin waves, resulting in actin ring formation (red) and cortical actin disruption within the wave. (**e**) PI(4,5)P_2_ waves (green) precede actin waves (red). (**f**) PI(3,4)P_2_ waves (yellow) follow actin waves. The area inside the actin wave becomes depleted of PI(4,5)P_2_ and enriched with PI(3,4)P_2_. (**g**) Representative image of propagating actin waves and membrane remodeling. The actin wave (red) is accompanied by PI(4,5)P_2_ (green) and PI(3,4)P_2_ (yellow) waves. These phosphoinositide changes recruit specific membrane and associated proteins (gray), remodeling the inner leaflet of the plasma membrane and generating a new membrane domain.

Macrophages engulf large particles (>0.5 μm) through various mechanisms, including phagocytic cup formation, the sinking mechanism, and trogocytosis [2,22]. Among these, Fcγ receptor (FcγR)-mediated phagocytosis, which involves phagocytic cup formation, has been the most extensively studied [1,4]. In this process, macrophages internalize targets via the phagocytic cup, a structure derived from the plasma membrane. The formation of the phagocytic cup is closely associated with actin cytoskeleton reorganization. Macrophages search for targets using membrane ruffles or filopodia. Upon encountering a target, they initiate engulfment by forming pseudopod-like membrane protrusions and disrupting the actin cortex at the collision site (Figure 1a). This disruption is facilitated by the inhibition of Arp2/3-dependent actin polymerization by coronin 1 and the severing of F-actin by gelsolin and cofilin [1]. The depletion of cortical actin alters the endomembrane structure, enhancing the lateral diffusion of membrane proteins, which promotes the clustering of FcγRs and interactions among other phagocytic receptors.

These morphogenetic events occur through the signal transduction cascade downstream of FcγR [1]. The stimulation of FcγR by IgG binding induces receptor clustering, triggering the tyrosine phosphorylation of the ITAM (immunoreceptor tyrosine-based activation motif) motif. The phosphorylated FcγR recruits secondary tyrosine kinases (e.g., Syk) and adaptor proteins (e.g., Nck) via their SH2 domains. Nck contains three SH3 domains that bind nucleation-promoting factors (NPFs) such as WASP and N-WASP. NPFs recruit Arp2/3 to the site of receptor clustering. Cdc42 and PI(4,5)P_2_ activate WASP/N-WASP, stimulating Arp2/3-mediated actin nucleation [23]. Additionally, Src-family kinase Hck can activate WASP by phosphorylating tyrosine 291. Consequently, activated Arp2/3 induces branched F-actin polymerization, leading to phagocytic cup elongation. Therefore, WASP and N-WASP, adaptor proteins that bind multiple signaling and cytoskeletal proteins, link the receptor activation to the reorganization of the actin cytoskeleton [24]. The two adaptor proteins are expressed in myeloid cells such as peritoneal macrophages [25] and osteoclast-like cells differentiated from primary bone marrow cells [26]. The assembly of these multivalent protein complexes promotes a phase transition to a gel-like state [27], which may mechanically facilitate efficient target grasping.

Macrophages also employ a supplementary structure, the constriction zone, during engulfment (Figure 1b). This substructure of the phagocytic cup comprises bundles of linear F-actin and a limited number of podosomes [2,28,30]. This organization enables the constriction zone to grasp targets by exerting forces in different vectors. Podosomes are Arp2/3-driven dynamic actin-rich adhesion sites involved in adhesion, migration, force generation, mechanosensing, and matrix degradation [31,32,33,34]. Podosomes in the phagocytic cup have been discussed elsewhere [35]; in this study, we summarize recent findings. Phagocytic podosomes were first reported as rings of F-actin puncta beneath advancing ruffled pseudopodia of the phagocytic cup. These puncta co-localized with ArpC2 and were surrounded by integrin β2, talin, vinculin, paxillin, and myosin IIA, similarly to conventional macrophage podosomes [30]. Notably, phagocytic podosomes exhibited shorter lifetimes than conventional podosomes, leading authors to term them “phagocytic podosomes”. Combining lattice light-sheet microscopy (LLSM) and microparticle traction force microscopy (MP-TFM) revealed that the arrangement of phagocytic podosomes at the internal rim of the phagocytic cup is advantageous for target gripping [28]. Super-resolution microscopy revealed that the actin core of phagocytic podosomes adopts an hourglass shape [36], distinct from the dome-like shape of conventional podosomes in clusters [34,37]. In summary, Arp2/3-dependent actin polymerization contributes to phagocytic cup formation at multiple stages.

## 3. Phosphoinositides and Membrane Polarization in Phagocytic Cup Formation

Phosphoinositides are concentrated in the inner leaflet of the plasma membrane, where their head groups interact with cytosolic and membrane proteins. These interactions facilitate the recruitment of soluble proteins to the membrane, leading to the assembly of signaling complexes. Since each phosphoinositide exhibits a distinct subcellular distribution, they play a crucial role in defining organelle identity [38]. For example, epithelial polarity, traditionally explained by the function and localization of polarity proteins such as PAR proteins [39,40], can also be described in terms of phosphoinositide metabolism, function, and localization [41,42]. Specifically, PI(3,4,5)P_3_ regulates the formation of the basolateral membrane in Madin–Darby canine kidney (MDCK) cells [43], whereas PI(4,5)P_2_ defines the apical membrane in MDCK cysts [44]. During phagocytic cup formation, phosphoinositide distribution undergoes significant changes [29,45]. In resting macrophages, PI(4,5)P_2_ is uniformly distributed along the inner leaflet of the plasma membrane. However, in the early stages of phagocytic cup formation, PI(4,5)P_2_ rapidly accumulates at the pseudopod, forming a distinct ring (Figure 1c). This PI(4,5)P_2_ ring persists until the phagocytic cup closes, leading to phagosome formation. Meanwhile, the area beneath the PI(4,5)P_2_ ring, corresponding to the body of the phagocytic cup, becomes enriched with PI(3,4,5)P_3_. Notably, the plasma membrane of the newly formed phagosome is rich in PI(3)P. These observations suggest that the dynamic localization and redistribution of phosphoinositides contribute to membrane polarization during phagocytic cup formation.

## 4. **The 2D Phagocytic Cup in Frustrated Phagocytosis**

Macrophages transform their plasma membrane into a pliable structure, the phagocytic cup, to internalize targets during phagocytosis. Masters et al. elucidated the organizational principles underlying this transformation by monitoring the molecular dynamics of actin waves using total internal reflection fluorescence (TIRF) microscopy [46]. They analyzed the behavior of fluorescently tagged molecules in frustrated phagocytosis using RAW 264.7 cells. Frustrated phagocytosis is a two-dimensional (*2D*) system in which macrophages plated on an IgG-coated glass surface form phagocytic cup-like structures. This system enables the high-spatiotemporal-resolution imaging of molecular dynamics. After five minutes of cell spreading, 10% of macrophages developed large actin rings (~10 µm in diameter) on their ventral membrane (Figure 1d). These rings contained actin regulators such as Arp2/3, N-WASP, and VASP and exhibited oscillatory behavior, alternating between expansion and contraction with a periodicity of 166 s. The expansion rate of the actin ring was at 2.4 µm/min, suggesting that it represents an actin wave.

To further characterize the molecular composition of these waves, the researchers examined the distribution of phosphoinositides and lipid-modifying enzymes. PI(4,5)P_2_ was enriched at the outer edge of the actin wave but depleted inside it (Figure 1e). In contrast, PI(3,4)P_2_ and diacylglycerol (DAG) were abundant within the wave (Figure 1f). Consistently with the suppression of PI(4,5)P_2_ synthesis, PI4P5-kinases—responsible for PI(4,5)P_2_ production—were depleted inside the actin wave, while the PI(4,5)P_2_ phosphatase OCRL1 was enriched in the same region. This resulted in a total depletion of PI(4,5)P_2_ in the cortical area enclosed by the actin wave. PI(3,4)P_2_ can be generated from PI(3,4,5)P_3_ via the action of 5′-phosphatases. Two such enzymes, synaptojanin-2 and SH2-domain-containing inositol 5′-phosphatase (SHIP2), localized to the inner edge of the actin wave. Notably, SHIP2 waves lagged behind actin waves by approximately 21 s. Interestingly, cortical actin and spectrin were depleted in the area enclosed by the actin wave (Figure 1d). Unexpectedly, this actin-depleted region exhibited high exocytic activity, as indicated by the release of GFP (green fluorescent protein)-GPI (glycosylphosphatidylinositol). These findings suggest that the propagation of circular actin waves remodels the inner leaflet of the plasma membrane, establishing a distinct membrane domain (Figure 1g). The formation of the 2D phagocytic cup in frustrated phagocytosis can thus be explained by the actin-wave-mediated compartmentalization of the plasma membrane and subsequent membrane remodeling. That the phosphoinositide distribution in the 2D phagocytic cup closely parallels that observed in the 3D phagocytic cup (Figure 1c) suggests similar polarizations also occur in 3D phagocytosis by macrophages.

## 5. Actin Waves

### 5.1. Chemical Waves

Actin waves are self-organized waves that ubiquitously form on the cortical surface of eukaryotic cells. They have been described under various names, including traveling waves [47], Hem-1 waves [48], and adhesive F-actin waves [49]. Given their diverse dynamics and molecular compositions, they are best categorized as chemical waves [50]. Actin waves play critical roles in numerous cellular processes, including migration, morphogenesis, pattern formation, polarization, intracellular protein transport, and phagocytosis. Readers are encouraged to refer to other reviews for an in-depth discussion of actin waves [51,52,53,54,55].

**Figure 2:**
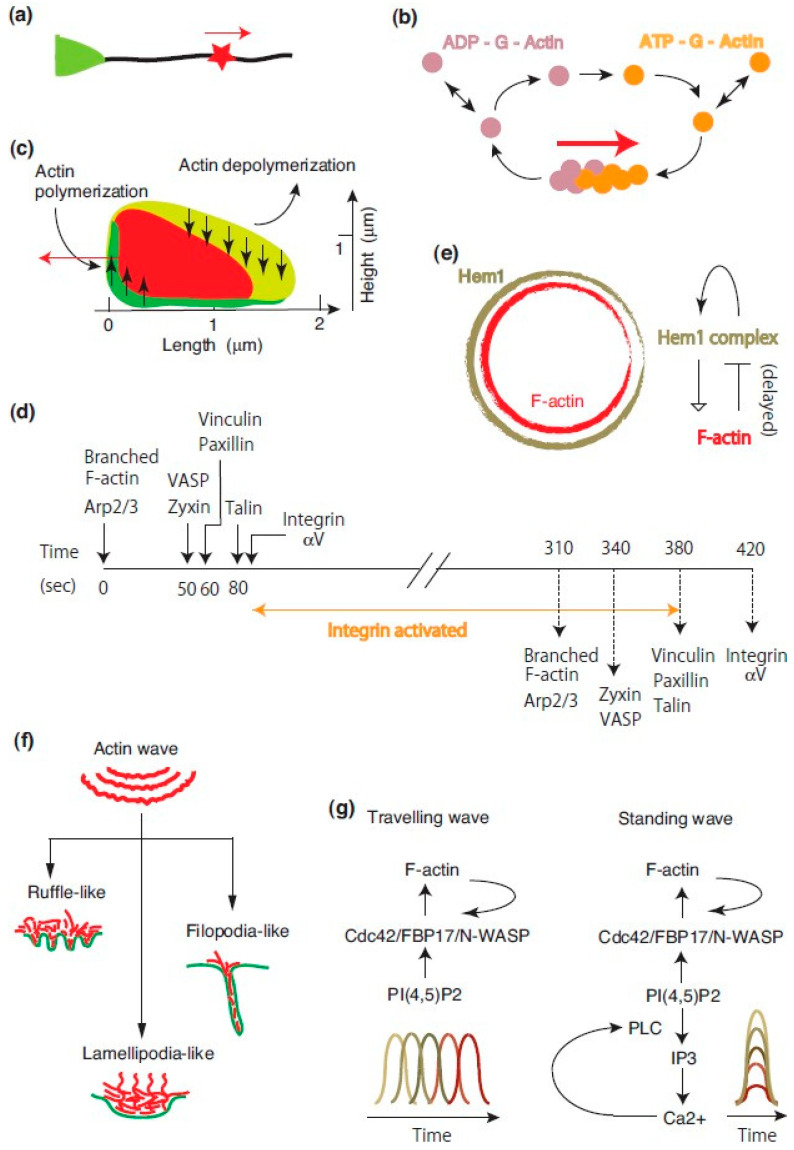
**Basics of actin waves.** (**a**) Actin wave in a hippocampal neuron. An actin patch (red star) propagates along an axon (green triangle). Adopted and modified from [52]. (**b**) Model of actin patch movement. Actin filaments within the patch migrate via directional polymerization and depolymerization. The red arrow indicates actin patch movement, while double arrows represent actin subunit exchange between the neutralizing and diffusible pools. Adopted and modified from [52]. (**c**) Cross-section of actin waves in *Dictyostelium* cells. Myosin-IB (green) accumulates at the wave front near the membrane, while coronin (yellow-green) localizes along the sloping roof of the wave. Vertical arrows indicate the up- and down-regulation of actin polymerization. The red arrow shows the wave propagation direction. Adopted and modified from [61,62]. (**d**) Chronology of adhesive F-actin waves. The formation and disassembly of adhesive F-actin waves follow a sequential pattern. Talin recruitment to the ventral F-actin wave (at 80 s) promotes integrin activation and ECM adhesion. Solid and dashed lines indicate wave assembly and disassembly, respectively. Adopted and modified from [49]. (**e**) Reciprocal interactions between Hem-1 and actin waves. Hem-1 waves precede actin waves (left). Membrane-bound Hem-1 promotes local F-actin polymerization via the WAVE2 complex (right). After a delay, F-actin displaces Hem-1 from the membrane. Adopted and modified from [48]. (**f**) Transformation of actin waves into protrusions. Actin wave formation is regulated by the signal transduction excitable network (STEN), involving small GTPases, phosphoinositides, and kinases. In *Dictyostelium* cells, STEN parameter changes generate various protrusions such as ruffles, lamellipodia, and filopodia. Adopted and modified from [59]. (**g**) Conversion of traveling waves to standing waves. FBP17, an Arp2/3 activator, exists in two states at the plasma membrane: horizontal and vertical waves. Ca^2+^ oscillations coupled with PI(4,5)P_2_ fluctuations induce vertical FBP17 waves, resulting in vertical actin wave formation. Adopted and modified from [63].

To simplify the discussion, we begin with one-dimensional actin waves. In rat hippocampal neurons undergoing axonal outgrowth, an actin patch moves from the axonal base to the tip (Figure 2a) [56]. This patch contains actin-associated proteins, regulators of F-actin treadmilling, and phospholipids. The directional assembly and disassembly of F-actin are thought to drive the movement of the actin patch (Figure 2b), thereby facilitating cargo transport along the axon.

Two-dimensional (*2D*) actin waves occur at the ventral membrane of various cells on flat surfaces. These waves can be classified as either *open* or *closed*. Open actin waves contribute to cell migration [57], cell polarity [58], and the formation of protrusive structures such as filopodia, lamellipodia, and ruffles [59]. In contrast, *closed* or *circular* actin waves participate in phagocytic cup formation during macrophage frustrated phagocytosis, as discussed earlier.

A notable example of closed actin waves occurs in *Dictyostelium* cells during 2D phagocytosis [60,61]. These waves spontaneously emerge at the ventral membrane, forming circular patterns in 2D while maintaining an ordered structure in 3D (Figure 2c). Myosin-IB, a single-headed myosin that binds membranes via its tail domain, localizes at the wavefront. Coronin, an Arp2/3 inhibitor, forms a sloping layer within the wave, while Arp2/3 is distributed throughout. This organization likely promotes wave propagation through net actin polymerization at the front and depolymerization at the rear.

The establishment of membrane polarity is suggested by the dynamic distribution of PI(3,4,5)P_3_ at the ventral membrane during the engulfment of elongated *Saccharomyces cerevisiae* particles (60). As the actin wave propagates, PI(3,4,5)P_3_ becomes enriched within the wave-enclosed area, maintaining its relative distribution despite wave expansion and retraction. The PI3-kinase inhibitor LY-294002 reversibly inhibits actin wave formation, indicating PI(3,4,5)P_3_’s role in wave dynamics. The authors concluded that the 2D phagocytic cup in *Dictyostelium* arises from cortical differentiation into distinct domains separated by actin waves, with wave patterns representing planar projections of the phagocytic cup [61]. This agrees with findings in macrophage frustrated phagocytosis [46], although macrophages utilize PI(3,4)P_2_ instead of PI(3,4,5)P_3_.

### 5.2. Organization of Actin Waves

Case and Waterman examined the dynamics of fluorescently tagged focal adhesion proteins—including F-actin, Arp2/3, zyxin, VASP, paxillin, vinculin, talin, and integrin αV—in U2OS cells, a human osteosarcoma cell line [49]. These focal adhesion proteins segregated into multiple waves, with some waves containing multiple components. The assembly and disassembly of these component waves collectively organized the adhesive F-actin wave (Figure 2d). The temporal sequence of protein assembly into the adhesive F-actin wave was as follows:

1. F-actin and Arp2/3;
2. Zyxin and VASP;
3. Paxillin and vinculin;
4. Talin;
5. Integrin αV.

Conversely, the order of disassembly was:

1. F-actin and Arp2/3;
2. Zyxin and VASP;
3. Paxillin, vinculin, and talin;
4. Integrin αV.

Pharmacological inhibitors of actin polymerization, latrunculin A, and cytochalasin D reduced the frequency of integrin αV waves. Conversely, functional-blocking antibodies targeting integrin αV also inhibited F-actin waves. Additionally, MnCl_2_ (an integrin activator) suppressed both actin and integrin αV waves. These findings suggest that integrin αV waves and F-actin waves are functionally coupled. The formation of adhesive F-actin waves requires cyclic engagement and disengagement of integrins with the extracellular matrix.

A feedback loop links Hem-1 waves to actin waves [48]. Hem-1/Nap1 is a subunit of the WAVE regulatory complex (WRC), a key actin regulator that interacts with small G-proteins, inositol phospholipids, and kinases [64]. Activated WRC promotes branched actin polymerization via Arp2/3 [65]. In HL-60 cells (a human neutrophil-like cell line), chemoattractant stimulation (fMLP) induces Hem-1 waves. These waves exhibit nonuniform distribution, velocity, and lifespan. Downstream effectors of Hem-1 signaling, such as active Rac, Arp2/3, and F-actin, spatially correlate with Hem-1 waves but are more diffusely distributed. Photobleaching experiments revealed rapid Hem-1 recovery before significant wave propagation, suggesting dynamic equilibration with the cytoplasmic pool. Latrunculin A treatment inhibited this rapid recovery, indicating that F-actin is necessary for Hem-1 recruitment from the cytosol. The authors proposed a model in which cycles of Hem-1 autoactivation and delayed F-actin-mediated inhibition generate Hem-1 waves (Figure 2e).

### 5.3. Actin Waves and Cellular Protrusions

Theoretical studies predict that Arp2/3-dependent treadmilling can generate self-organized actin waves and patches [66]. Actin waves form circular rings, arc shapes, and more complex patterns in *Dictyostelium* cells [67]. Various cellular protrusions, including filopodia, ruffles, and lamellipodia, emerge from propagating cortical waves [59].

A theoretical framework divides actin waves into two substructures:

- The signal transduction excitable network (STEN), including PIP_2_, Ras, and PKB/Akt;
- The cytoskeleton excitable network (CEN), containing F-actin and Rac.

These networks form feedback loops, with PKB/Akt linking STEN to CEN and STEN activity modulated by CEN feedback. Artificially altering actin wave parameters induces distinct protrusions in *Dictyostelium*, mimicking filopodia, ruffles, and lamellipodia (Figure 2f). This suggests that actin waves serve as a unifying mechanism for various protrusive structures.

### 5.4. Behavior of Actin Waves

All actin waves described above propagate horizontally. However, horizontal waves can transform into vertical standing waves through coupling with intracellular Ca^2^⁺ oscillations (Figure 2g) [63]. In RBL-2H3 cells (a tumor mast cell line), antigen stimulation induces actin waves that originate from Cdc42/FBP17/N-WASP/PI(4,5)P_2_ waves. FBP17, a membrane-associated activator of N-WASP and Arp2/3, exists in two distinct physical states at the plasma membrane: a traveling (horizontal) wave and standing oscillations (vertical wave). Antigen stimulation not only generates actin waves but also triggers Ca^2^⁺ release from intracellular stores. The calcium-dependent hydrolysis of PI(4,5)P_2_ by phospholipase C establishes a feedback loop involving intracellular Ca^2^⁺, PI(4,5)P_2_, and PI(3,4,5)P_3_ oscillations. Interestingly, FBP17 and PI(4,5)P_2_ at the plasma membrane oscillate in perfect synchrony with cytosolic Ca^2^⁺ oscillations, but in opposite phases. This coupling of oscillations drives FBP17 standing oscillations, ultimately leading to the formation of standing actin waves.

How do cells perceive flat surfaces in their environment? A study using *Dictyostelium* cells confined between two parallel surfaces—a cover glass on the ventral side and a wedged microcantilever on the dorsal side—provides insights [68]. These cells generate propagating waves of PI(3,4,5)P_3_ and F-actin. Under confinement, they exhibit surface-switching behavior with a periodicity of 2–5 min. When an actin wave forms on one surface, wave formation on the opposite surface is suppressed. Moreover, wave initiation and propagation show a strong preference for the opposite surface (*n* = 378/428). Frequency spectra obtained from both the glass and the cantilever surfaces are highly similar, suggesting that fluctuations between the two surfaces are coupled. The authors proposed that actin wave formation is regulated by an autonomous oscillatory switch mechanism. Such dynamic properties of actin waves may enable cells to navigate and adapt to diverse 3D environments in both physiological and pathological contexts. While the behavior and functional significance of actin waves in 3D environments remain largely unexplored, further investigation promises to yield significant discoveries in cell biology.

## 6. Bone Resorption Apparatus

Osteoclasts undergo large-scale intracellular reorganization to facilitate bone resorption [12,13]. The plasma membrane of a bone-resorbing osteoclast is highly polarized, forming distinct functional domains: the functional secretory domain, basolateral membrane, sealing zone, and ruffled border. The ruffled border can be further divided into the fusion zone and uptake zone. However, the mechanisms underlying this membrane polarization remain unclear. In the following sections, we compare the formation of bone resorption machinery in osteoclasts with phagocytic machinery in macrophages. Specifically, we examine whether the sealing zone and ruffled border in osteoclasts correspond to the actin wave and the inner region of the actin wave observed in two-dimensional phagocytosis, respectively.

### 6.1. Sealing Zone

**Figure 3:**
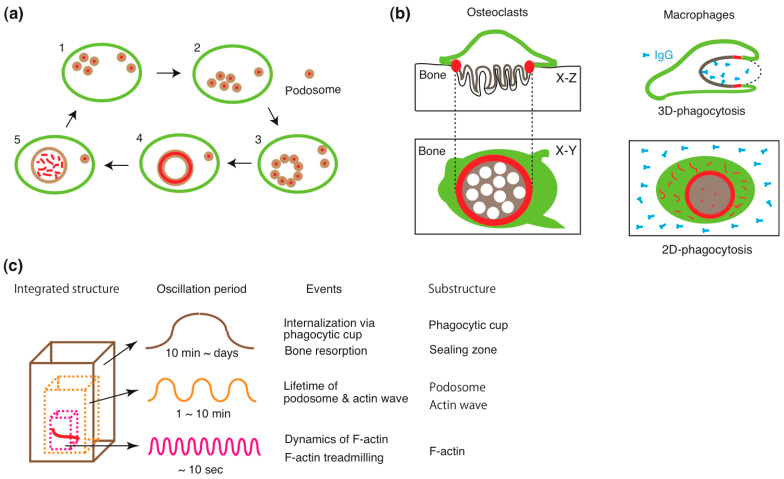
**Self-organized structures in phagocytes.** (**a**) Assembly patterns of podosomes in osteoclasts during the bone resorption cycle. 1: Podosomes are either dispersed as dots or assembled into rosettes at the paramarginal area of migrating osteoclasts. 2: Podosomes coalesce at the prospective resorption site, forming a broad band. 3: The podosomes further organize into a broad ring with a clear, distinguishable arrangement. 4: The F-actin ring condenses to form the sealing zone in actively resorbing osteoclasts. 5: Following bone resorption, the F-actin ring disperses, indicating the post-resorptive stage of osteoclasts. In typical podosomes observed in stages 1, 2, and 3, the actin core (red) is surrounded by an adhesion domain (beige). In stages 4 and 5, adhesion domain proteins redistribute to form a larger, continuous ring. Adopted and modified from [70]. (**b**) Structural apparatus for osteoclastic bone resorption and macrophage phagocytosis. Left: Cross-sectional (upper) and horizontal (lower) views of a bone-resorbing osteoclast. The osteoclast attaches to the bone through the sealing zone (red). Inside the sealing zone, the plasma membrane transforms into a ruffled border (brown) for resorption. Right: Cross-sectional (upper) view of a macrophage internalizing a target. The macrophage engulfs the target with a phagocytic cup (brown) and secures it tightly using a constriction zone (red). Horizontal section (lower) shows a macrophage undergoing frustrated phagocytosis on an IgG-coated glass surface, where a closed actin wave (red) is formed. Within the wave, localized phosphoinositide distribution leads to cortical actin disruption and membrane remodeling (brown). (**c**) An integrated oscillation model. Phagocytes utilize dynamic, self-organized oscillatory structures to perform their functions. These structures consist of three hierarchical oscillatory substructures, categorized by oscillation frequency. The innermost layer involves high-frequency F-actin treadmilling, providing the driving force for the formation of larger structures. The intermediate layer consists of mesoscale structures such as podosomes and actin waves, assembled through dynamic actin polymerization and depolymerization. The outermost layer consists of large, integrated structures such as the sealing zone, ruffled border, phagocytic cup, and constriction zone, maintained by the underlying oscillatory processes. The oscillatory dynamics of the smaller substructures energize and sustain the integrated structures.

Podosomes, self-organized actin-rich structures, are present at the plasma membrane and exhibit distinct assembly patterns during the in vitro differentiation of macrophages into osteoclasts [69]. Osteoclasts display five podosome assembly patterns throughout the bone resorption cycle (Figure 3a) [70,71]. The bone-resorbing osteoclast adopts a ring-like podosome assembly pattern, known as the sealing zone (Type 4 in Figure 3a). The sealing zone consists of a dense F-actin ring, with podosomal adhesion proteins distributed along its inner and outer peripheries. Some osteoclasts possess multiple sealing zones, which expand through ring enlargement and fusion [72,73]. Osteoclasts terminate bone resorption by disassembling the sealing zone, making it a key marker of active osteoclasts.

The sealing zone shares structural and functional similarities with the constriction zone of the macrophage phagocytic cup (Figure 3b). First, the sealing zone consists of a dense actin meshwork interspersed with podosomes [74,75,76], similarly to the constriction zone, which contains phagocytic podosomes and bundles of F-actin cables [30]. Phagocytic podosomes generate vertical (protrusive) forces against their target through Arp2/3-dependent branched actin polymerization, while linear F-actin cables generate circumferential contractile forces via interactions with myosin II (Figure 1b). These two force-generating mechanisms enable the phagocytic cup to securely grasp its target [2,28,77,78]. In osteoclasts, podosomes are thought to exert vertical forces through F-actin treadmilling, while linear actin filaments linking podosome heads contribute to collective podosome behavior and the formation of functional podosome islets [79]. Such mechanisms may facilitate the firm attachment of osteoclasts to the complex bone surface.

Second, actin waves may serve as the origin of both the sealing zone and the constriction zone. The transformation of actin waves into the phagocytic cup has been well documented [46,60,61], whereas the origin of the sealing zone remains unclear. The focal actin cloud [73] and actin patches have been proposed as precursors of the sealing zone [80,81]. We previously hypothesized that actin waves transform into the sealing zone [14]. Supporting this hypothesis, osteoclast-like cells lacking Hem-1, a key component of actin waves [48], fail to form sealing zones on calcium phosphate surfaces [82].

Finally, both the sealing zone [12,83] and the constriction zone [30,84,85] function as diffusion barriers, restricting lateral molecular movement. This function is essential for osteoclasts to create an isolated resorption compartment, yet its precise mechanism remains elusive. Integrins appear to play a crucial role in establishing this barrier in both the sealing zone [86] and the phagocytic cup [30,84,85]. Supporting this, osteoclasts deficient in kindlin-3, a master regulator of integrins, form defective, leaky sealing zones [87].

### 6.2. Ruffled Border

Osteoclasts degrade bone by secreting hydrogen ions and digestive enzymes through the ruffled border membrane onto the bone surface [12]. Lysosomal vesicles transport vacuolar-type proton ATPase (V-ATPase) and cathepsin K to the ruffled border [88], leading some researchers to describe the ruffled border as an “external lysosome” [89]. Currently, the ruffled border is considered a key trafficking center for endocytic, secretory, transcytotic, and autophagic pathways [90,91]. The formation and maintenance of the ruffled border depend on the dynamic fusion and fission of membrane vesicles. Once bone resorption is complete, osteoclasts rapidly disassemble the ruffled border.

A comparable process occurs in macrophages, where the phagocytic cup transforms into a phagosome via sequential fusion with early and late endosomal and lysosomal vesicles [4]. Within the phagolysosome, macrophages degrade their target, a process theoretically analogous to osteoclastic extracellular digestion. Notably, human neutrophils release lysosomal materials extracellularly when positioned on a non-phagocytosable surface [92]. Similarly to the ruffled border, the phagosome disappears after target degradation.

### 6.3. Coupling of the Sealing Zone with the Ruffled Border

Frustrated phagocytosis suggests that the compartmentalization and membrane remodeling induced by actin waves are coupled processes (Figure 1g and Figure 3b). If this coupling follows a causal relationship, defects in compartmentalization should be accompanied by abnormal membrane remodeling. This hypothesis is supported by genetic studies of osteoclasts. Osteoclast-like cells lacking integrin β3 fail to form the podosome belt on glass and exhibit abnormally thick, blunted ruffled borders on bone [93]. Similar abnormalities are observed in osteoclasts deficient in Hem-1 [82], pp60c-Src [94], and Vav3 [95], as well as in osteoclast-like cells with Arp2/3 knockdown [96]. Pharmacological inhibition of phosphatidylinositol-3 kinase with wortmannin also produces comparable defects [97].

Conversely, osteoclasts with a normal sealing zone but a defective ruffled border have been identified in incisor-absent rats [98], as well as in patients with mutations in PLEKHM1 (pleckstrin homology domain-containing family M, with RUN domain, member 1) [99] and SNX10 (sorting nexin 10) [100]. Similar phenotypes occur in osteoclast-like cells with Rab7 [101] or ATP6ap1 (Ac45) knockdown, or those treated with U18666A, an inhibitor of late endosomal/lysosomal trafficking [102]. These findings reinforce the notion that vesicle trafficking is necessary for ruffled border formation but not for sealing zone assembly. To date, no osteoclasts have been reported with a normal ruffled border but lacking the sealing zone, suggesting that the sealing zone is a prerequisite for ruffled border formation and that their development is a coupled process (Figure 3b).

The coupling between the sealing zone and ruffled border suggests that bone resorption apparatus formation involves membrane polarization. Epithelial polarity is established through the epithelial polarity program (EPP), which is orchestrated in response to extracellular cues and governs apico-basal membrane polarity and intercellular junctions via cytoskeletal rearrangement and polarized trafficking [40]. While macrophages and osteoclasts lack intercellular junctions, they develop functionally similar structures: the constriction zone in the phagocytic cup and the sealing zone in osteoclasts. These structures act as diffusion barriers akin to epithelial tight junctions. Interestingly, epithelial junctional proteins such as ZO-1 have been detected in podosomes of smooth muscle cell lines (A7r5 cells) [103] and primary human brain microvascular endothelial cells [104]. This raises an intriguing question: Could actin waves play a role in establishing epithelial polarity?

## 7. Organizational Principle: Fractal Integration of Self-Organized Oscillatory Substructures

In this study, we consider the structural features originating from actin waves. The formation and maintenance of actin waves depend on the treadmilling of F-actin, a process characterized by the continuous addition and release of G-actin. Given this oscillatory nature, we focus on the periodic behavior of these self-organized substructures. To represent their dynamics, we cite collective values derived from experimental observations.

In *Dictyostelium* cells, actin waves propagate at a speed of 6 μm/min, with an F-actin lifetime of 20 s. Actin fluorescence recovers to half-maximal levels within 4 s after photobleaching [62]. The corresponding values for Hem-1 waves in HL-60 cells are 6 μm/min, 23 s, and 6.4 s, respectively [48]. The sealing zone in human osteoclast-like cells on bone comprises three distinct actin fluorescence components with oscillation periods of 7, 25, and 100 s [79]. In macrophage podosomes, actin fluorescence oscillates with periods of 30 and 44 s [105], while atomic force microscopy (AFM) measurements detect two stiffness oscillation components (6 and 32 s) in the podosomes [106]. These results indicate that actin-associated behavior in actin waves and podosomes exhibits oscillatory dynamics on a timescale of tens of seconds. Moreover, coupling actin waves with other waves leads to the formation of larger assemblies. The lifetimes of podosomes within the sealing zone of rabbit osteoclasts range from 2 to 12 min [107]. The expansion and shrinkage of podosome rosettes exhibit oscillation periods of 4 and 2 min, respectively [108]. The lifetimes of podosomes in podosome clusters, podosome rings, and podosome belts of osteoclast-like cells are approximately 2 min [69]. Similarly, the actin cores of podosomes in human dendritic cells persist for 5–15 min [109]. In RAW264.7 macrophages, podosome lifetimes at podosome clusters and phagocytic cups are 100 s and 50 s, respectively [30]. The assembly and disassembly cycle of adhesive F-actin waves takes 7 min [49], while the surface-switching behavior of actin waves in *Dictyostelium* oscillates with periods of 2–5 min [68]. To estimate the timescale of collective structures, we consider the duration required for the completion of functional activity. Small sealing zones (<7 μm in diameter) persist for approximately 8 min, whereas larger ones (>14 μm) can last for hours to days [72,73]. The internalization of a target by the phagocytic cup ranges from 10 min to several hours. These values can be categorized into three subclasses based on oscillation periods (Figure 3c), which may correspond to underlying substructures. The integration of these substructures forms a unified system, which we refer to as the “integrated oscillation model”.

The classification of oscillation periods may decode fundamental organizational principles. Since substructures share oscillatory dynamics, their integration follows a fractal pattern in this model. The elementary oscillation unit is F-actin treadmilling, in which the addition and release of G-actin to and from F-actin occurs on a timescale of approximately one second under physiological salt conditions in vitro [110,111,112]. However, these reactions proceed more rapidly in cells due to the involvement of actin-regulatory proteins. The substructures constructed directly from F-actin and its derivatives oscillate with a period of about 10 s. Larger substructures exhibit lower oscillation frequencies. The new components such as actin-binding or -accessory proteins may be added to the substructure via chemical waves. The lifetime of larger substructures such as actin waves and podosomes is about several minutes. The participation of actin-binding proteins modulates the shape and dynamics of the oscillatory substructures, contributing to the diversity of the final integrated structures. Since the expression of actin-binding proteins depends on cell types and species, differences in treadmilling kinetics may partially account for the diversity of invadosomes and protrusive structures. The duration of bone resorption cycles varies significantly, with pit-forming osteoclasts operating on a timescale of several hours and trench-forming osteoclasts persisting for several days [113]. Thus, integrated structures can persist for over five orders of magnitude longer than the timescale of elementary actin reactions. F-actin treadmilling may be a key strategy for living organisms to dynamically adapt and extend their functional structures in space and time.

The complexity of macrophage phagocytosis is widely recognized [1]. The efficiency and mechanisms of phagocytosis vary depending on cell type, differentiation state, and target properties. Similar challenges arise when studying podosomes/invadopodia [34] and actin waves [51,54]. These dynamic structures, composed of F-actin, integrins, and phospholipids, form at the interface between the cytosol, plasma membrane, and extracellular matrix. Their functions are regulated by integrin receptor activity, and they are collectively referred to as the “integrin adhesome” [114]. Integrin adhesomes are organized through the stratification of functionally distinct modules [115,116,117]. In focal adhesions, these modules are arranged into three vertical layers: (1) an integrin signaling layer containing integrin, FAK, and paxillin; (2) a force transduction layer containing talin and vinculin; and (3) an actin regulatory layer containing F-actin, VASP, and α-actinin. The actin regulatory layer is further subdivided into three sublayers [118]. The adhesion domain of podosomes exhibits a similar stratification [117]. These structures appear to be organized through the physical segregation of interactome modules. Notably, parts of the phagocytic cup and bone resorption apparatus contain integrin adhesomes. Investigating how such modular stratification integrates into the oscillation model remains an intriguing avenue for future research.

From a mechanical perspective, self-organized actin patterns at the plasma membrane are independent of macroscopic mechanical properties [119]. This suggests that specialized membrane structures can undergo large-scale rearrangements with minimal impact on overall cellular mechanics. The phagocytic cup, podosomes, and sealing zones are formed on demand [2,31], requiring rapid assembly and disassembly while minimizing disruption to other cellular structures. The fractal architecture emerging from Arp2/3-mediated oscillatory waves may provide an optimal solution to these structural demands.

## 8. Perspectives

This review examines macrophage phagocytosis and osteoclastic bone resorption from a structural perspective. Actin waves organize phagocytic cup-like structures in frustrated phagocytosis by macrophages, reminiscent of the sealing zone and ruffled border formation in osteoclasts. Circular actin waves may serve as the origin of both the sealing zone and the constriction zone of the phagocytic cup. The ordered assembly and disassembly of component waves drive self-organization, while subtle changes in F-actin treadmilling parameters shape various actin-based superstructures. Phosphoinositide waves flanking actin waves regulate F-actin polymerization, remodeling cortical actin and endomembrane structures. Together, these processes compartmentalize the plasma membrane, contributing to membrane polarization in epithelial cells. Consequently, macrophage phagocytosis and osteoclastic bone resorption become aspects of cell polarity. Studying these phenomena from multiple perspectives—including differentiation, signal transduction, cytoskeletal and membrane remodeling, and self-organization—will enhance our understanding and may lead to novel therapeutic strategies for diseases related to macrophage and osteoclast dysfunction.

Self-organization is a fundamental principle in biology. In osteoclasts, it governs the formation and assembly of podosomes, which serve as substructures of the sealing zone. Thus, self-organization is integral to bone resorption. Similarly, actin waves are self-organized structures involved in diverse cellular processes. Our research identified actin waves in various podosome assemblies in osteoclast-like cells, leading to the hypothesis that actin waves contribute to sealing zone formation. However, this model did not account for the ruffled border, another essential bone resorption machinery. In this review, we expanded the hypothesis by proposing that circular actin wave propagation remodels membrane compartments. This refined model links sealing zone formation with ruffled border development, unifying the understanding of osteoclastic bone resorption.

A similar organizational principle applies to the formation of 2D phagocytic cups in *Dictyostelium* cells and frustrated phagocytosis in macrophages. We propose that actin wave-driven phagocytic structures integrate oscillatory substructures, with oscillatory behavior stemming from F-actin treadmilling. Other cell types may employ comparable principles to construct specialized structures and execute their unique functions.
